# Depth-stratified boreal peatland microbiomes reveal recurring community organization and methane-cycling potential

**DOI:** 10.1093/femsec/fiag111

**Published:** 2026-10-01

**Authors:** Tong Liu, Jacob Smeds, Mats Nilsson, Mats Öquist, Stefan Bertilsson

**Affiliations:** Department of Forest Ecology and Management, Swedish University of Agricultural Sciences (SLU), Umeå, 90183, Sweden; Department of Aquatic Sciences and Assessment and Science for Life Laboratory, Swedish University of Agricultural Sciences (SLU), Uppsala, 75007, Sweden; Department of Forest Ecology and Management, Swedish University of Agricultural Sciences (SLU), Umeå, 90183, Sweden; Department of Forest Ecology and Management, Swedish University of Agricultural Sciences (SLU), Umeå, 90183, Sweden; Department of Forest Ecology and Management, Swedish University of Agricultural Sciences (SLU), Umeå, 90183, Sweden; Department of Aquatic Sciences and Assessment and Science for Life Laboratory, Swedish University of Agricultural Sciences (SLU), Uppsala, 75007, Sweden

**Keywords:** peatlands, methanogenesis, microorganisms, vertical stratification, metagenome-assembled genomes, 16S rRNA gene

## Abstract

Microbial communities in peatlands vary with depth, but most studies have been limited in spatial extent or replication, constraining assessment of general community patterns. We characterized microbial community structure and metabolic potential across replicated depth profiles in seven boreal peatlands in Sweden. Combining 16S rRNA gene sequencing and shotgun metagenomics, we identified recurring vertical stratification across sites. Microbial richness declined with depth, and core taxa including Rice Cluster II, Terriglobales, Subgroup 2, *Roseiarcus, Methylocystis*, and Candidatus *Solibacter* were shared across locations. Functional gene profiles revealed depth-related reorganization of carbon-cycling potential, with hydrolytic and fermentative functions relatively more abundant near the surface and acetogenic and methanogenic pathways more prominent in deeper anoxic horizons. Gene-centric analyses indicated an important contribution of Rice Cluster II-related methanogens to hydrogenotrophic methanogenesis, while co-occurrence networks identified a depth-conserved syntrophic module centered on this lineage. Environmental variables showed depth-specific associations with microbial communities. Despite geographic separation and site-specific variation among peatlands, recurring depth-resolved microbial assemblages and functional profiles were observed, suggesting that common environmental constraints associated with peat development contribute to broad patterns of microbial community organization and carbon-cycling potential.

## Teaser

Conceptual illustration of depth-related microbial organization and methane-cycling potential in northern peatlands. From left to right, the figure depicts representative microbial taxa along the peat depth profile and their putative ecological or methane-cycling roles (Created with BioRender.com).

## Introduction

Wetlands significantly influence global climate regulation by means of carbon sequestration as well as storage of biomass and detrital organic matter (Lolu et al. 2020). These ecosystems also deliver essential ecological services, including mitigation of eutrophication, provision of diverse habitats for aquatic and terrestrial organisms, hydrological buffering that reduces flooding risk, and groundwater replenishment. Although wetlands serve as important carbon sinks, they also emit greenhouse gases, particularly methane (CH₄), generated under anaerobic conditions that prevail in deeper, water-saturated layers (Salimi et al. 2021). Northern peatlands, constituting ∼44% of global wetlands, are characterized by long-term accumulation of partially decomposed organic material, forming substantial peat deposits that have exerted a cooling effect on Earth’s climate over millennia (Frolking et al. 2011). Peatlands exhibit distinct vertical environmental gradients, with upper peat layers characterized by higher oxygen availability and faster decomposition rates, shifting to deeper anaerobic layers where fermenting microbial activity, linked to methane production, becomes prominent. Microbial communities in peatlands are in general strongly shaped by such steep vertical gradients, resulting in distinct compositional stratification along peat depth profiles (Wang et al. 2024). These patterns have important implications for organic matter decomposition, nutrient cycling, and methane dynamics, as well as the establishment of plant functional types (PFT). For instance, aerobic microbial groups dominate upper peat layers while methanogenic archaea and other anaerobic taxa become increasingly prevalent at greater depth. Although depth-related microbial stratification has been described in several peatland studies, it remains unclear to what extent the taxonomic composition, magnitude, and functional organization of these depth-dependent patterns are shared across geographically distinct boreal peatlands. Most previous studies have focused on individual peatlands and limited replication, also limiting our ability to evaluate the generality and reproducibility of depth-dependent microbial organization across larger spatial scales. (Zhou et al. 2017, Wang et al. 2024, Shen et al. 2025).

While 16S rRNA gene data have advanced our understanding of taxonomic diversity, they offer limited resolution for inferring metabolic potential and often fail to detect uncultured or deeply branching lineages due to primer bias and incomplete reference databases (Hug et al. 2016). Genome-resolved metagenomic studies have begun to address these gaps by recovering novel microbial genomes from thawing permafrost and peat environments, revealing uncharacterized clades involved in methanogenesis and carbon cycling (Woodcroft et al. 2018). However, most of these efforts have focused on individual sites with minimal replication, restricting the ability to generalize from single systems to entire peatland landscapes.

In this study, we address these limitations by combining depth-resolved vertical sampling with broad multi-site replication and genome-resolved metagenomic analysis. We sampled seven boreal peatlands spanning an extensive latitudinal gradient across Sweden (57–63°N; see Fig. 1 in Smeds et al. 2025). Three replicate peat cores were collected per site and sectioned into five depth intervals; after exclusion of three contaminated cores, 105 samples were retained for microbial community analysis.

**Figure 1:**
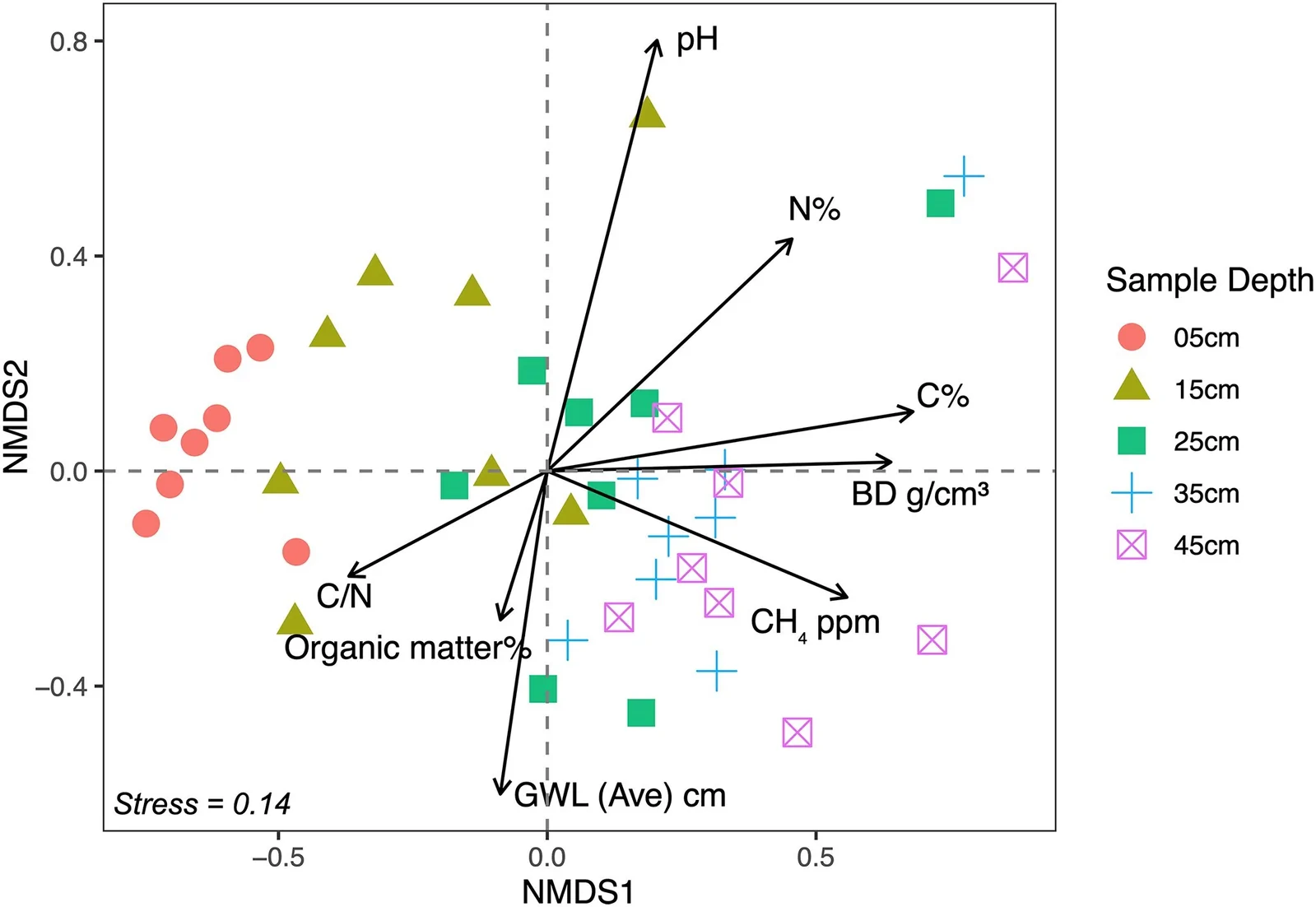
Non-metric Multidimensional Scaling (NMDS) plot of microbial communities based on 16S rRNA gene data. Points are colored according to peat depth, and environmental factors associated with microbial community structures are illustrated as vectors, where the length and direction of vectors indicate the degree of correlation with community data. Abbreviations correspond to porewater pH (pH), bulk density (BD; g cm⁻³), CH₄ concentration in porewater (CH₄; ppm), organic matter content (“Organic matter%”; mass-%), carbon content (C%; mass-%), nitrogen content (N%; mass-%), C/N ratio (C/N), and average groundwater level (GWL; cm below the peat surface).

Based on previous studies of peatland stratification, we hypothesized that, although both peat depth and geographic location would contribute substantially to microbial community variation, microbial communities would show recurring depth-related organization across geographically distinct boreal peatlands. To test this hypothesis, we aimed to (1) characterize microbial diversity, composition, and metabolic potential across peatland depth profiles; (2) identify persistent core, transient, and potential keystone taxa associated with depth-dependent community organization; and (3) examine how microbial community patterns relate to environmental gradients and hydrological conditions across peatlands.

## Method and materials

### Site description

We selected seven boreal peatlands spanning a latitudinal gradient of ∼750 km across Sweden (57–63°N), representing both minerogenic and ombrogenic boreal peatlands with oligotrophic to mesotrophic nutrient status (Smeds et al. 2025). Two separate locations within the Ånnsjön mire (AN) complex were sampled and treated as independent sites, resulting in a total of eight sampling sites. Dominant vegetation consisted primarily of Sphagnum mosses together with varying abundances of sedges, dwarf shrubs, and other mire-associated vascular plants. Detailed site characteristics, including peatland classification, dominant vegetation, pH, groundwater level, methane concentration, and geographic coordinates, are provided in Supplemental file S4. Three replicate peat cores were collected from each site and sectioned into five depth intervals. To minimize vegetation-related variability, sampling was restricted to lawn communities.

### Peat sample collection and characterization

The three cores from each site were sampled ∼10 to 15 m apart using a modified 50 cm long circular stainless-steel corer with a 16 cm inner diameter (Clymo 1988). A total of 24 peat cores were collected from eight sampling sites across seven peatlands. Three cores (MT2, MT3, and OM3) were excluded from microbial analyses because of contamination, leaving 21 cores for downstream analysis. Samples were collected at 5 cm, 15 cm, 25 cm, 35 cm, and 45 cm depth using sterilized tweezers, homogenized in ziploc bags before aliquots were frozen in 2 ml cryo tubes by immersing in liquid nitrogen and subsequently stored long-term at −80°C until further analysis. For environmental variables, the peat cores were transported to the lab within 4 h of sampling and stored at −18°C. The frozen peat cores were sub-sectioned in a 4°C lab to prevent melting and preserve integrity. To reduce the impact of vegetation for comparisons between peat layers, the uppermost 4 cm of the peat cores were first removed, i.e. the samples were sliced and labelled as 5, 15, 25, 35, and 45 cm from the mire surface. To measure groundwater level (GWL), submerged groundwater loggers (Solinst Levellogger Junior; Solinst Ltd, Canada) were installed within 1 m of each sampling point and recorded water level continuously from mid-July to the end of August 2021. This period encompassed the peat sampling campaign for both microbiological and chemical analyses. Atmospheric pressure was recorded separately and subtracted to calculate true groundwater level. All depth measurements in this study (5–45 cm) refer to vertical distance from the peat surface. After core slicing, the peat discs were dried for ∼72 h in a 70°C oven until constant weight. Dry samples were immediately placed in a silica gel desiccator to prevent moisture absorption during cooling for subsequent weight determination (Mettler Toledo AG204 scale). The dry bulk density of each peat soil sample was determined by dividing the dry mass of the sample by its corresponding volume. Environmental variables used in this study, including porewater methane concentration, pH, organic matter content, and elemental composition (C and N), were obtained from the accompanying peatland characterization study based on the same sampling campaign (Smeds et al. 2025), where detailed descriptions of sample collection, laboratory analyses, and analytical procedures are provided.

### Microbial community analysis

#### Total DNA extraction and quantification

Approximately 200 mg of frozen peat material from each depth-specific subsample collected in the field using sterilized tweezers and immediately preserved in liquid nitrogen (105 samples in total, derived from 21 uncontaminated cores × five depths) was used for total genomic DNA extraction with the DNeasy PowerSoil Kit (Qiagen, Valencia, CA, USA), according to the manufacturer protocol. DNA concentration in the extracts was quantified with a Qubit 3.0 Fluorometer using Qubit 1X dsDNA Broad Range (BR) Assay Kits (Invitrogen, Thermo Fisher Scientific, Waltham, MA, USA).

#### 16S rRNA gene amplicon analysis

A 16S rRNA gene amplicon library for Illumina sequencing was prepared using primers 341F (CCTACGGGNGGCWGCAG) and a modified version of the reverse primer 805R (GACTACHVGGGTATCTAATCC) (Herlemann et al. 2011), here designated 805NR (GACTACNVGGGTATCTAATCC). This primer set have 95% and 89.8% coverage of bacteria and archaea, respectively, in the SILVA SSU r138.2 (RefNR) database (mismatches = 2, tested on 2025–02–24), indicating broad coverage of both bacterial and archaeal communities (Supplemental file S1). The detailed PCR procedure and sequence library preparation steps have been previously described (Sinclair et al. 2015). Briefly, PCR was performed using Q5 High-Fidelity DNA Polymerase with an initial denaturation at 98°C for 3 min, followed by 20 cycles of 98°C for 10 s, 48°C for 30 s (annealing), and 72°C for 30 s, with a final extension at 72°C for 2 min. The annealing temperature used (48°C) has been tested to enable amplification of sequences with up to three mismatches to the primers (Sipos et al. 2007) and thus provide sufficient permissiveness to support our *in silico* coverage estimate based on two mismatches. Next-generation amplicon sequencing was performed in-house using MiSeq (Illumina) technology with Reagent Kit v3, generating 2 × 300 bp paired-end reads. The raw data was analyzed with the open-source bioinformatics pipeline DADA2 (version 1.16) as implemented in R (version 4.0.2) (Callahan et al. 2016). Forward and reverse primers were trimmed before merging paired reads, and the resulting sequences were cut to lengths of 270 and 160 bp, respectively, with quality thresholds of maxEE = (2, 2) and truncQ = 2, according to in silico calculation by FIGARO (Weinstein et al. 2019). Taxonomic profiles were assigned based on amplicon sequence variants (ASVs) using the rRNA database SILVA, release 138.2 (Quast et al. 2013). The sequencing raw data can be accessed via the National Centre for Biotechnology Information (NCBI) database (BioSample accession SAMN38645172 under BioProject PRJNA1048504).

Illumina sequencing of 16S rRNA gene libraries from 105 peat samples generated a total of 2 841 890 raw reads. After filtering, paired-end merging, and chimera removal, 859 624 high-quality sequences remained, averaging 8187 sequences per sample (range: 3491–21 291). Taxonomic classification was robust, with fewer than 4% of merged reads per sample remaining unclassified at the kingdom level. Detailed information regarding read filtering procedures, forward and reverse read quality, and sequencing depth is provided in Supplemental file S2.

A substantial reduction in read numbers occurred predominantly during paired-end merging using the DADA2 pipeline. This reduction was primarily due to poor sequence quality toward the 3′ ends of the reverse reads (R2), a known limitation of the MiSeq (Illumina) platform. To evaluate potential impacts of this loss, we initially tested a relaxed quality filtering threshold (Q ≥ 20). However, this adjustment did not significantly affect the results of downstream multivariate analyses or microbial community composition. Consequently, we adopted a stringent quality filtering threshold (Q ≥ 30), accepting greater read loss but ensuring high-quality, reliable data that reflect true biological variation.

#### Shotgun metagenome assembly and genome recovery

Peatland samples from the Ånnsjön mire (AN) were selected as a representative boreal peatland for whole genome shotgun sequencing because the site included the most comprehensive sampling effort and did not exhibit outlier behavior in microbial community composition compared with the other investigated peatlands (see Results). For metagenomic sequencing, DNA extracts from the three replicate cores at each depth were pooled prior to library preparation to generate composite metagenomic samples. Library preparation followed the Illumina DNA PCR-Free Library Prep, Tagmentation protocol (Document #1000000086922 v03). Sequencing was performed at the SNP&SEQ Technology Platform in Uppsala, Sweden, using the NovaSeq 6000 system with a single SP flowcell and v1.5 sequencing chemistry (Illumina Inc.), generating 150 bp paired-end reads. The raw metagenomic sequencing reads are available through the National Center for Biotechnology Information (NCBI) Sequence Read Archive (SRA) under BioProject PRJNA1048504. The recovered metagenome-assembled genomes (MAGs) have also been deposited in the same BioProject.

Raw metagenomic reads were analyzed using the open-source SqueezeMeta pipeline (v1.6.5) in co-assembly mode (Tamames and Puente-Sánchez 2019). Reads from all metagenomic samples were co-assembled using MEGAHIT (v1.2.9), after which quality-filtered reads from each sample were mapped back to the assembled contigs using the default Bowtie2-based mapping procedure implemented in SqueezeMeta. Mapping statistics, including the number of mapped reads, mapping percentage, and total mapped bases, are provided in Supplemental file S8. Genome bins were reconstructed from the co-assembled metagenome using the MetaBAT2 (v2.15) binning algorithm implemented in SqueezeMeta.

Genome completeness and contamination were estimated using CheckM2 (v1.1.0), which applies a machine learning framework trained on high-quality reference genomes to predict genome quality from genomic features rather than relying solely on lineage-specific marker genes (Chklovski et al. 2023). Recovered genomes were classified as high-quality (HQ; ≥90% completeness and ≤5% contamination), medium-quality (MQ; ≥50% completeness and ≤10% contamination), or low-quality (LQ). A total of 665 genome bins were recovered, of which 207 HQ and MQ MAGs were retained for downstream analyses and database archiving.

Taxonomic classification of the retained MAGs was performed using GTDB-Tk (v2.5.2) with the Genome Taxonomy Database (GTDB release R226) (Chaumeil et al. 2022). GTDB-Tk identifies a standardized set of conserved bacterial (bac120) or archaeal (ar53) marker genes, aligns these markers to the GTDB reference database, and infers genome taxonomy by phylogenetic placement within the GTDB reference tree. Taxonomic assignments were generated using the classify_wf workflow and were used consistently throughout downstream analyses and for MAG submission to NCBI.

### Statistical analyses

Permutational multivariate analysis of variance (PERMANOVA) was conducted using the *adonis2* function in the vegan R package (version 2.6–4) to assess which environmental variables significantly explained variation in microbial community composition (based on Bray–Curtis dissimilarities, 999 permutations). Vector fitting was performed using the *envfit* function (also from vegan) to identify and visualize the correlation between environmental gradients and NMDS ordination space. Student *t-test* was performed in R (version 4.3.1) to analyze variation in pore water methane concentrations across the samples. Correlation analyses among environmental variables were performed using the *corrplot* package (version 0.92). Samples were rarefied using *rrarefy* of vegan (version 2.6–6.1) to the lowest read count before alpha diversity analysis. ASVs contributing > 1% of the total sequence reads in the combined dataset were used for co-occurrence network analysis (*RCy3* and *igraph* package in R). Spearman’s rank correlations were calculated between each pair of ASVs, and p values were corrected by the Benjamini–Hochberg correction to control for false discovery rate upon multiple comparisons (Haynes 2013). Only significant and strong correlations (*P* < 0.05, Spearman’s correlation coefficient (ρ) > 0.5) were included in the co-occurrence network. Degree, betweenness, and closeness centrality indices were calculated to evaluate the co-occurrence network structure and identify potential keystone microorganisms (Berry and Widder 2014). The co-occurrence network figure was plotted using *Cytoscape* (version 3.7.2). To visualize functional gene abundances along the peat profile, KEGG Orthologs (KOs) involved in anaerobic carbon transformation pathways were identified based on KEGG annotations. TPM values (length-normalized reads per million) for each KO were obtained from the SqueezeMeta pipeline (v1.6.5) (Supplemental file S3). For each KO, mean and maximum TPM across all samples were calculated, and the union of the top 150 KOs ranked by mean TPM and the top 150 ranked by maximum TPM was retained as a representative subset for downstream functional profiling. To complement the whole-metagenome KO-based functional profiling, we examined the putative metabolic capabilities of representative microbial lineages detected in the 16S rRNA gene dataset by linking them to taxonomically assigned ORFs in the metagenomic annotation table. For each representative lineage, ORFs were selected based on taxonomic assignments at the genus or, where necessary, higher taxonomic levels, and their associated KEGG Orthologs were summarized across peat depths. Because these associations were based on taxonomic matching of metagenomic ORFs rather than recovered genomes, they represent taxon-guided functional inference rather than direct genome-resolved functional assignment. All data processing and visualization were conducted in R (version 4.3.1) using the *tidyverse* and *ggplot2* packages.

## Results

### Vertical and spatial patterns in peat chemistry

As presented in our recent study focusing specifically on peat chemistry (Smeds et al. 2025), there was no clear systematic trend across peatland locations in chemical variables such as bulk density, organic matter content, carbon and nitrogen concentrations, and methane levels, however, distinct vertical gradients in these properties were evident (Supplemental file S4).

### 16S rRNA gene-Based community analyses

#### Community structure and environmental correlates

16S rRNA gene amplicon analysis identified a total of 46 phyla and 428 unique genera. Among these, 11 phyla and 42 genera exceeded 3% relative abundance in at least one sample. Microbial community composition varied primarily along the vertical depth profile and showed strong associations with environmental context, while no consistent clustering was observed among peatlands or between replicate cores within sites (Fig. S1). Still, PERMANOVA showed significant effects of both sampling depth (*R*² = 0.175, *P* = 0.001) and peatland location (*R*² = 0.215, *P* = 0.001) on microbial community composition. Their interaction was also significant (*R*² = 0.229, *P* = 0.001), indicating that the magnitude or composition of depth-related community shifts varied among peatland locations. Together, depth, location, and their interaction explained 61.9% of the total community variation. Non-metric multidimensional scaling (NMDS) analysis revealed distinct depth-dependent clustering of microbial communities clearly separating surface (5 cm), intermediate (15–25 cm), and deeper layers (35–45 cm) (Fig. 1). Surface peat samples (5 cm) formed a distinct cluster clearly separated from other depths. Intermediate-depth samples (15–25 cm) showed transitional assemblages, while deeper samples (35–45 cm) were more variable but distinct from surface communities (Fig. 1).

Several peat chemistry variables were significantly associated with microbial community composition, as confirmed by both PERMANOVA and vector fitting using *envfit*. Among the variables displayed in the NMDS ordination (Fig. 1), CH₄ concentration (*R*² = 0.43, *P* = 0.001), bulk density (*R*² = 0.41, *P* = 0.002), carbon content (*R*² = 0.36, *P* = 0.001), nitrogen content (*R*² = 0.28, *P* = 0.015), C/N ratio (*R*² = 0.20, *P* = 0.047), pH (*R*² = 0.24, *P* = 0.021), and average groundwater level (GWL; *R*² = 0.37, *P* = 0.001) showed statistically significant correlations with community structure. These gradients corresponded with depth-related changes in microbial composition, where surface samples (5–15 cm) were associated with higher C/N ratios and lower bulk density, while deeper samples (35–45 cm) were aligned with increased CH₄ concentrations, higher nitrogen content, and greater bulk density.

Across the peat profile, correlations involving methane and related chemical variables varied with depth (Fig. S2). CH₄ concentrations were negatively correlated with pH at all sampled depths (Pearson’s *r* = −0.38 to −0.10) and positively correlated with bulk density (*r* = 0.07–0.73), with the strongest positive correlation at 5 cm. Bulk density was positively correlated with carbon and nitrogen contents but negatively correlated with the C/N ratio at all depths. Carbon and nitrogen contents were positively correlated throughout the profile (*r* = 0.49–0.77). Acetate and sulfate exhibited weak correlations with CH₄ that varied in direction across depths.

#### Depth-driven shifts in dominant microbial taxa

Alpha diversity based on 16S rRNA gene data showed a marked decline from 5 to 25 cm, after which all diversity indices remained relatively stable with no significant differences between 25, 35, and 45 cm (Fig. S3). This decrease in richness and evenness from the upper to deeper peat layers was accompanied by a pronounced shift in microbial community composition along the peat profile. This compositional change, consistently observed across the sampled peatlands, was largely driven by shifts in the relative abundance of Halobacteriota and several lineages within Acidobacteriota, Planctomycetota, Pseudomonadota, and Thermodesulfobacteriota. From 5 to 45 cm, Halobacteriota and Thermodesulfobacteriota, represented by the uncultivated family Rice Cluster II, and *Desulfobacca*, respectively, increased markedly from <1% at 5 cm to >20 and 9% at 45 cm (Fig. 2). In contrast, multiple Alphaproteobacterial genera within Pseudomonadota (including *Methylocystis, Methylocapsa*, Xanthobacteraceae, *Methylocella, Roseiarcus*, and related lineages) collectively decreased from 22.2 ± 2.2% in the 5–15 cm layers to 8 ± 5.8% at 45 cm. Surface-enriched taxa such as *Aquisphaera* (Planctomycetota), *Acidothermus*, Acidimicrobiaceae, *Bryobacter* (Actinomycetota), *Gemmataceae* (Planctomycetota), and the WD2101 soil group were detected at higher relative abundance in the 5–15 cm layers but declined sharply below 25 cm. In deeper layers (25–45 cm), Anaerolineaceae (Chloroflexota) and *Methanoregula* (Halobacteriota) were consistently present at low relative abundance, generally below 3% across peatlands, with occasional peaks of 4–5% in individual deep samples. Several Acidobacteriota groups (*Terriglobales*, Subgroup 2, *Occallatibacter* and Candidatus *Solibacter*) were consistently abundant across all depths (typically 5%–15%).

**Figure 2:**
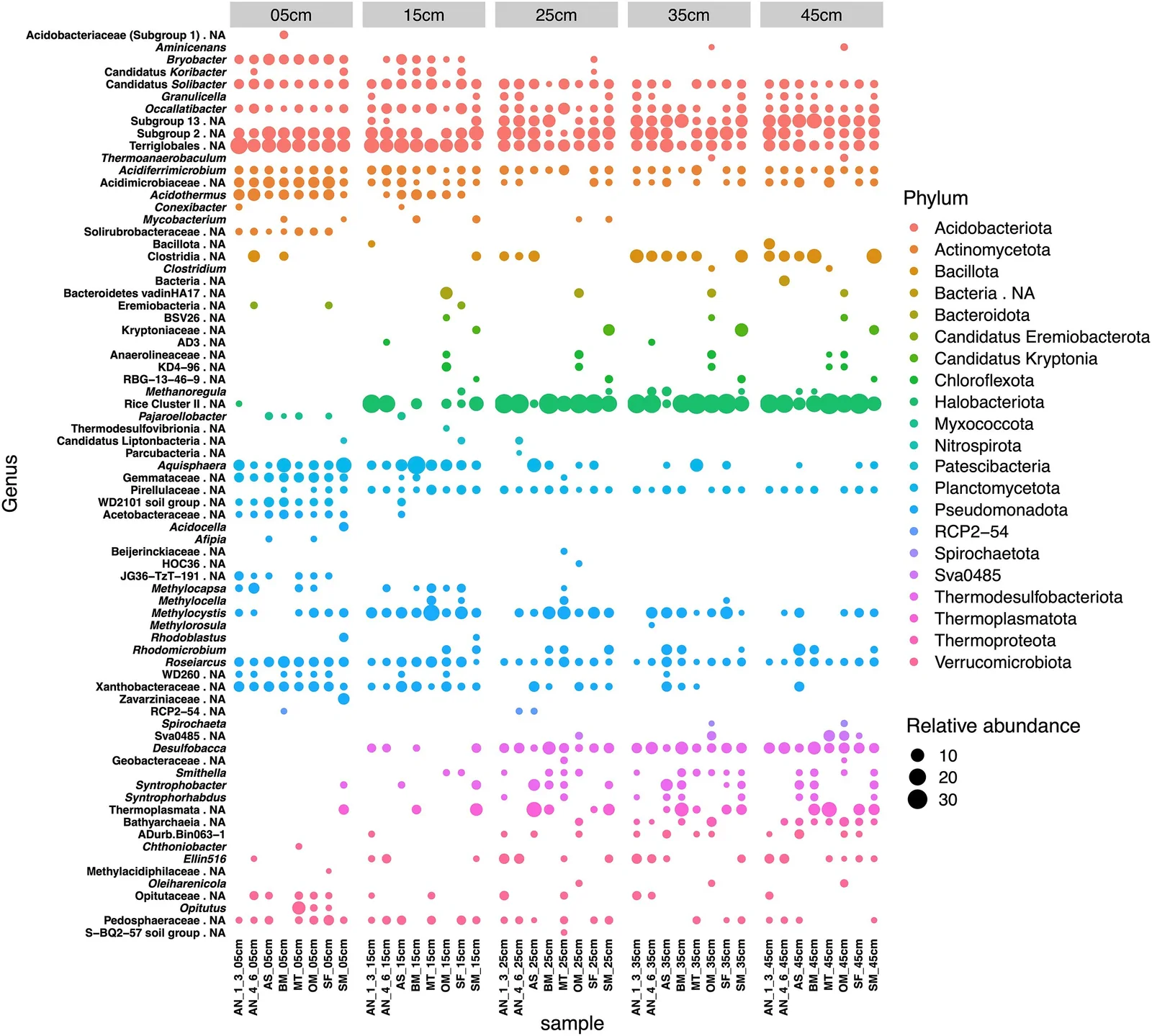
Relative abundance of genera within the microbial community based on the average ASV reads of 16S rRNA genes from peatland samples grouped according to sampling depth. Genera are color-coded according to phylum (right legend). Circle size represents the relative abundance of each identified genus. The samples at each depth were ordered from north to south (left to right). When a specific genus could not be robustly assigned, the closest classified taxonomic level is listed. Genera with relative abundance > 1% in at least one sample is depicted.

The observed shifts in relative abundances at the phylum level was mainly seen in the top 15 cm of the peat core, at the range of the average GWL changes (Supplemental file S5). Spatial shifts in relative abundances were also observed for more highly resolved taxonomic lineages. For example, within the phylum Pseudomonadota, *Methylocystis* increased from 5 to 15 cm but declined markedly at deeper layers (25–45 cm), whereas *Roseiarcus* remained present across all depths with relative abundances around 2% (Fig. 2). Within *Acidobacteriota, Bryobacter* was restricted to the upper 5–15 cm layers and occurred at <1% below 15 cm. In contrast, Candidatus *Solibacter* remained consistently abundant around 3% throughout the 5–45 cm profile. Subgroup 13 was detected at low abundance (<1%) at the upper layers and increased to around 8.5% at the bottom layer (Fig. 2).

#### Core and transient microbial community structure along the peat profile

Following established definitions (Shade and Handelsman 2012, Liu et al. 2022), genera detected in more than 80% of samples were classified as “core”, and those detected in fewer than 20% as “transient”. To identify these groups across peat depths, we examined occurrence–abundance relationships based on 16S rRNA gene amplicon data from 5–45 cm (Fig. 3). For this analysis, all individual peat cores were treated as independent samples, without merging within peatland × depth combinations, resulting in 105 samples distributed across five depth layers.

**Figure 3:**
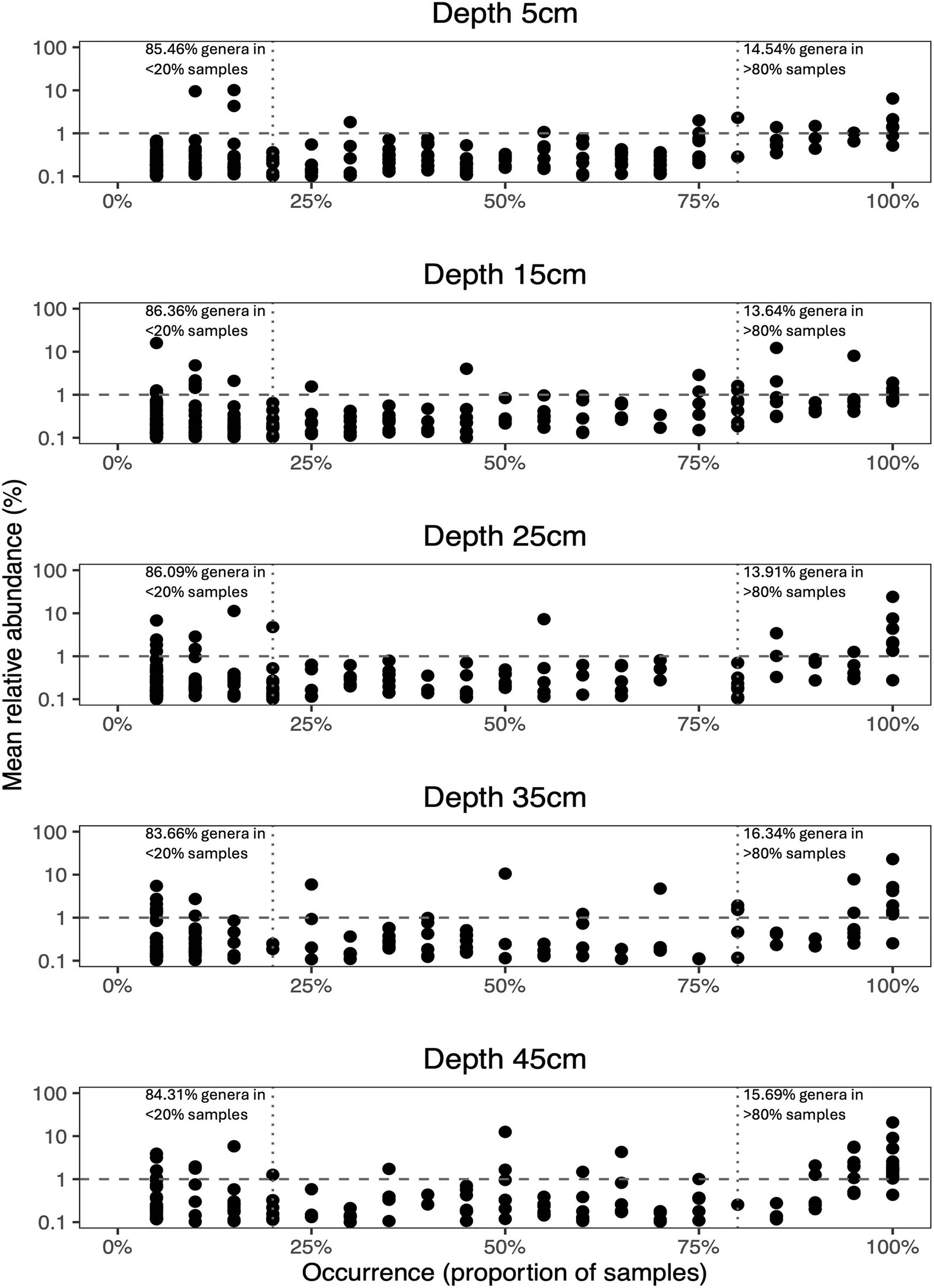
Occurrence versus mean relative abundance of genera across five peat depths (5–45 cm), based on 16S rRNA gene amplicon data. Dashed horizontal lines indicate 1% relative abundance in the overall microbial community. Dashed vertical lines indicate thresholds for “core” (>80% occurrence) and “transient” (<20% occurrence) genera. Labels within each panel summarize the percentage of genera falling into these categories.

Across all detected genera, the core–transient structure remained consistent along the depth gradient, with 83%–86% of genera classified as transient (<20% occupancy) and 14%–16% as core (>80% occupancy) at each depth (Fig. 3). No significant depth-related trend in core proportion was detected (Cochran–Armitage test, Z = 0.65, *P* = 0.52). Restricting the analysis to genera with a mean relative abundance >1% increased the relative contribution of core taxa (67–88% across depths), yet still revealed no significant linear trend with depth (Z = −1.00, *P* = 0.32) (Fig. 3). In contrast, dominance increased significantly with depth, as the cumulative relative abundance of the top five genera per sample rose from 43% at 5 cm to ∼57% at 35–45 cm (Spearman *ρ* = 0.90, *P* = 0.037) (Supplemental file S6), while α-diversity indices (Observed richness, Shannon, and Simpson) declined significantly from 5 cm to deeper layers, with no significant differences among 15–45 cm (Fig. S3).

At 5 cm, the top five most abundant core genera included Terriglobales (11.7%), Acidobacteriota Subgroup 2 (7.1%), *Aquisphaera* (6.4%), Acidimicrobiaceae (4.7%), and *Acidothermus* (4.4%). At 15 cm, Rice Cluster II became dominant (12.3%), followed by Terriglobales (10.6%), *Aquisphaera* (8.0%), Subgroup 2 (7.0%), and *Methylocystis* (5.6%). At 25 cm, the top five core assemblages were dominated by Rice Cluster II (24.0%), Subgroup 2 (7.6%), Terriglobales (6.0%), Acidobacteriota Subgroup 13 (4.9%), and *Desulfobacca* (4.4%), and this pattern persisted at 35–45 cm, with *Desulfobacca* and Subgroup 13 slightly increasing to 5.7% and 9.1%, respectively (Supplemental file S6).

#### Microbial network structure and keystone associations

To assess the ecological connectivity and potential functional associations between dominant microbial taxa in boreal peatlands, we performed a co-occurrence network analysis based on 16S rRNA gene amplicon data from all peat samples (Fig. 4). While the preceding section identified core genera based on their consistent presence across samples, the network analysis provides a complementary perspective by highlighting taxa that are tightly embedded in microbial co-occurrence networks. As noted in a previous study, core and keystone taxa represent distinct ecological dimensions, the former defined by ubiquity, and the latter by their structural influence in microbial networks (Liu et al. 2022).

**Figure 4:**
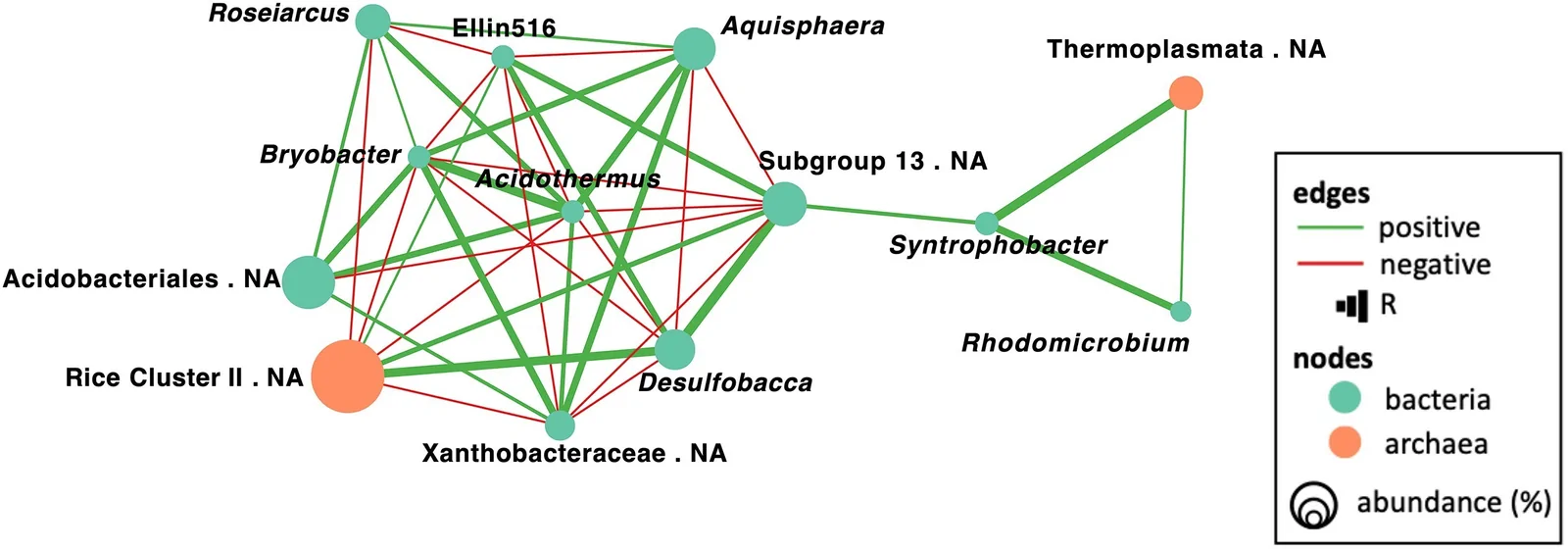
Co-occurrence network analysis based on the correlation of the relative abundance of amplicon sequence variant (ASV) reads at genus level or the closest classified taxonomic level (with each node containing at least three ASVs and >1% relative abundance) across all peatlands. Bacterial and archaeal taxa are shown as nodes colored in green and orange, respectively. Node size reflects average relative abundance. Each edge indicates a significant correlation (*P* < 0.05, R > 0.5) between node pairs, with green edges representing positive correlations and red edges indicating negative correlations. Edge thickness corresponds to the strength (R value) of the correlation.

The network revealed a prominent module centered around the uncultivated archaeal lineage Rice Cluster II (family Methanoflorentaceae), which strongly co-occurred with *Desulfobacca* and an unclassified Acidobacteriae Subgroup 13 member. In addition, *Desulfobacca*, an unclassified member of Xanthobacteraceae, *Acidothermus*, and *Aquisphaera*, displayed high degree and connectance, low betweenness, qualifying them as putative keystone taxa based on network topology (Supplemental file S7) (Berry and Widder 2014).

### Metagenomic reconstruction and functional profiling via MAGs

#### MAG Recovery and Assembly Quality

Shotgun metagenome sequencing of the composite peat samples from the Ånnsjön mire (AN) yielded ∼33–146 million raw paired-end reads per sample. After quality control, 57.5%–96.5% of the reads were successfully mapped back to the co-assembled contigs, corresponding to ∼5.0–22.0 Gbp of mapped sequence per sample (Supplemental file S8), indicating that the metagenome assembly captured a large proportion of the microbial communities. Co-assembly of all metagenomic samples generated an assembly of ∼7.17 Gbp, comprising 9.71 million contigs with an N50 of 880 bp, and resulted in the recovery of 665 metagenome-assembled genomes (MAGs). Genome quality was assessed using CheckM2 according to the MIMAG criteria. Among the recovered MAGs, 41 were classified as high-quality (HQ), 166 as medium-quality (MQ), and 458 as low-quality (LQ) genomes (Supplemental file S8). The 207 HQ and MQ MAGs were retained for downstream analyses and public deposition. Taxonomic classification of the retained MAGs was performed using GTDB-Tk (GTDB release R226), providing standardized genome-based taxonomic assignments across both bacterial and archaeal genomes. The retained MAGs comprised 179 bacterial and 28 archaeal genomes, with Acidobacteriota, Chloroflexota, Verrucomicrobiota, Actinomycetota, Thermoproteota, Desulfobacterota, and Pseudomonadota representing the most frequently recovered phyla (Supplemental file S8). These GTDB classifications were used consistently throughout all downstream analyses.

#### Comparison of 16S rRNA gene and MAG-derived community profiles

Community composition inferred from HQ and MQ MAGs shared several dominant phyla with the 16S rRNA gene amplicon profiles, but differed in their relative abundances and depth-related patterns (Fig. 5). Acidobacteriota was the dominant bacterial phylum in both datasets at most depths, accounting for 40.2%–42.9% of the 16S rRNA gene community and 17.8%–41.5% of the MAG-derived profiles. In both approaches, Pseudomonadota was most abundant in surface peat and had lower relative abundance in the deepest layer, decreasing from 23.5% to 6.2% in the 16S dataset and from 22.1% to 5.6% in the MAG-derived profiles. Actinomycetota decreased from 17.2% to 5.9% in the 16S dataset, whereas its MAG-derived relative abundance varied non-monotonically with depth, reaching 18.5% at 15 cm, declining to 9.4% at 25 cm, and increasing to 14.7% at 45 cm.

**Figure 5:**
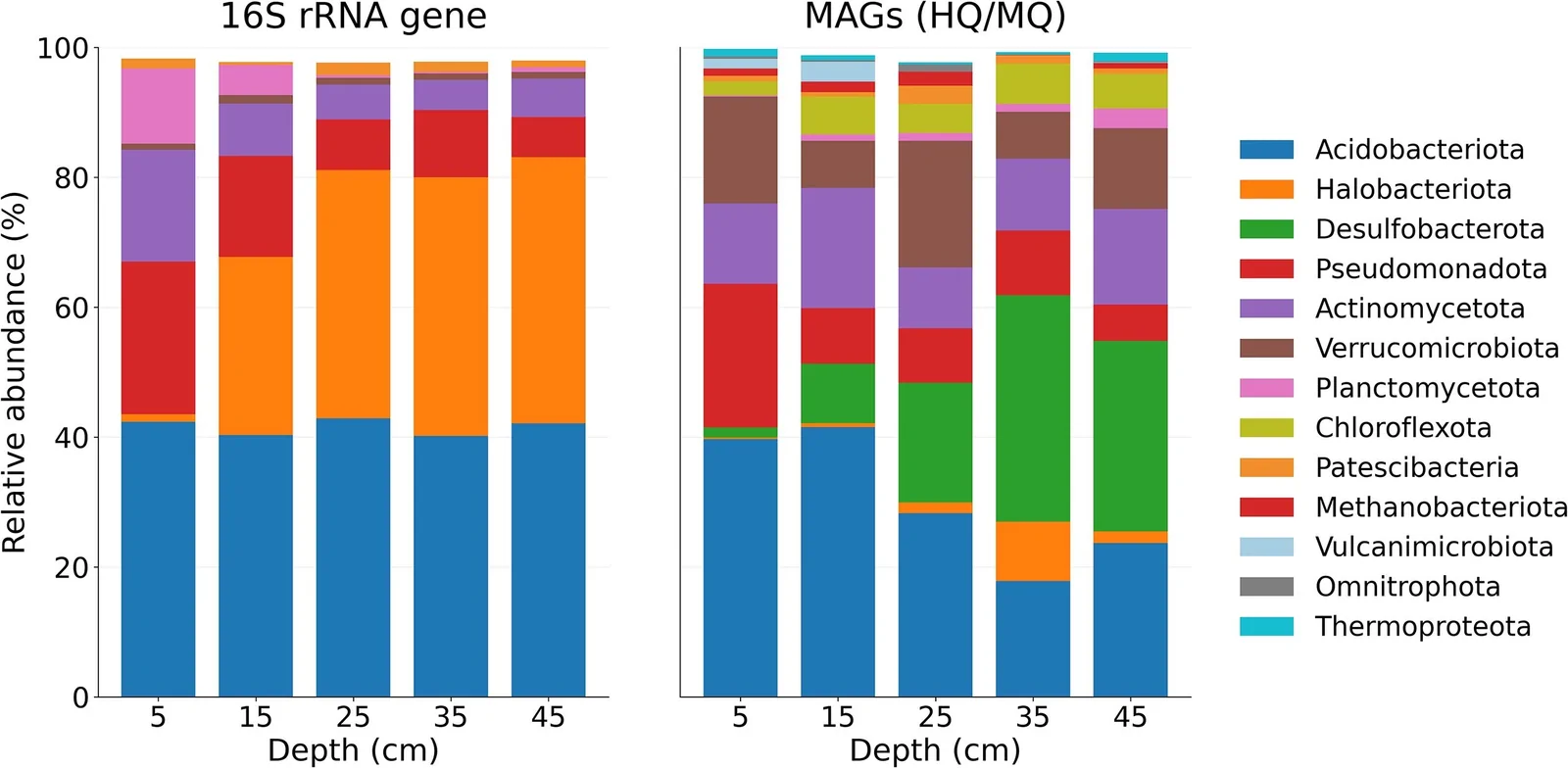
Comparison of phylum-level microbial community composition inferred from 16S rRNA gene amplicon sequencing and high- and medium-quality metagenome-assembled genomes (HQ/MQ MAGs) across the peat depth profile in the Ånnsjön peatland (AN). Relative abundances are shown for samples collected at five depths (5–45 cm). For the genome-resolved profiles, read counts assigned to each MAG were divided by its total assembled length, normalized to the sum across the 207 retained HQ/MQ MAGs within each sample, and then summed by phylum. Only phyla representing ≥1% relative abundance in at least one sample are shown. Relative abundances were not renormalized after omitting low-abundance phyla.

The largest discrepancy between the two approaches was observed for Halobacteriota. In the 16S rRNA gene dataset, Halobacteriota increased markedly from 1.2% at 5 cm to 41.0% at 45 cm, whereas its relative abundance in the HQ/MQ MAG-derived profiles ranged from 0.3% to 9.1%, peaking at 35 cm before decreasing to 1.8% at 45 cm. Conversely, Desulfobacterota, Chloroflexota, Thermoproteota, and Verrucomicrobiota were consistently more abundant in the MAG-derived profiles. Desulfobacterota increased from 1.5% at 5 cm to 34.9% at 35 cm and remained abundant at 45 cm (29.3%). Chloroflexota and Thermoproteota reached 5.4% and 1.4%, respectively, at 45 cm, while Verrucomicrobiota peaked at 19.5% at 25 cm. Patescibacteria, which represented 0.4%–1.9% of the 16S rRNA gene community, were also represented in the HQ/MQ MAG-derived profiles, ranging from 0.7% to 2.8%, with a maximum at 25 cm.

Despite quantitative differences in the relative abundance of several bacterial and archaeal phyla, the HQ/MQ MAG-derived profiles recapitulated the major depth-related shifts observed in the 16S rRNA gene dataset.

#### Depth-resolved functional profiles of the whole metagenome

Whole-metagenome functional profiling based on KEGG Ortholog (KO) annotations revealed clear depth-resolved shifts in the relative abundance of genes associated with major anaerobic carbon cycling processes across the peat profile (Fig. 6a). Genes associated with hydrolytic processes accounted for ∼10%–15% of the total TPM assigned to the selected carbon cycling functional categories near the surface and decreased progressively with depth. Genes associated with fermentation represented the largest functional category at all depths, accounting for ∼60%–80% of total TPM, with a steady decline from 5 to 25 cm followed by a slight recovery in deeper layers. Genes associated with acetogenesis remained relatively stable across the profile, contributing roughly 8%–12% at all depths with only minor fluctuations. In contrast, genes associated with methanogenesis increased markedly with depth, rising from below 2% at 5 cm to over 15% at 35–45 cm. These patterns collectively indicate a transition from surface-dominated hydrolysis and fermentation toward proportionally greater reductive carbon fluxes in deeper, more anoxic strata, consistent with the depth-dependent shifts in microbial community composition inferred from both the 16S rRNA gene dataset and the genome-resolved HQ/MQ MAG profiles.

**Figure 6:**
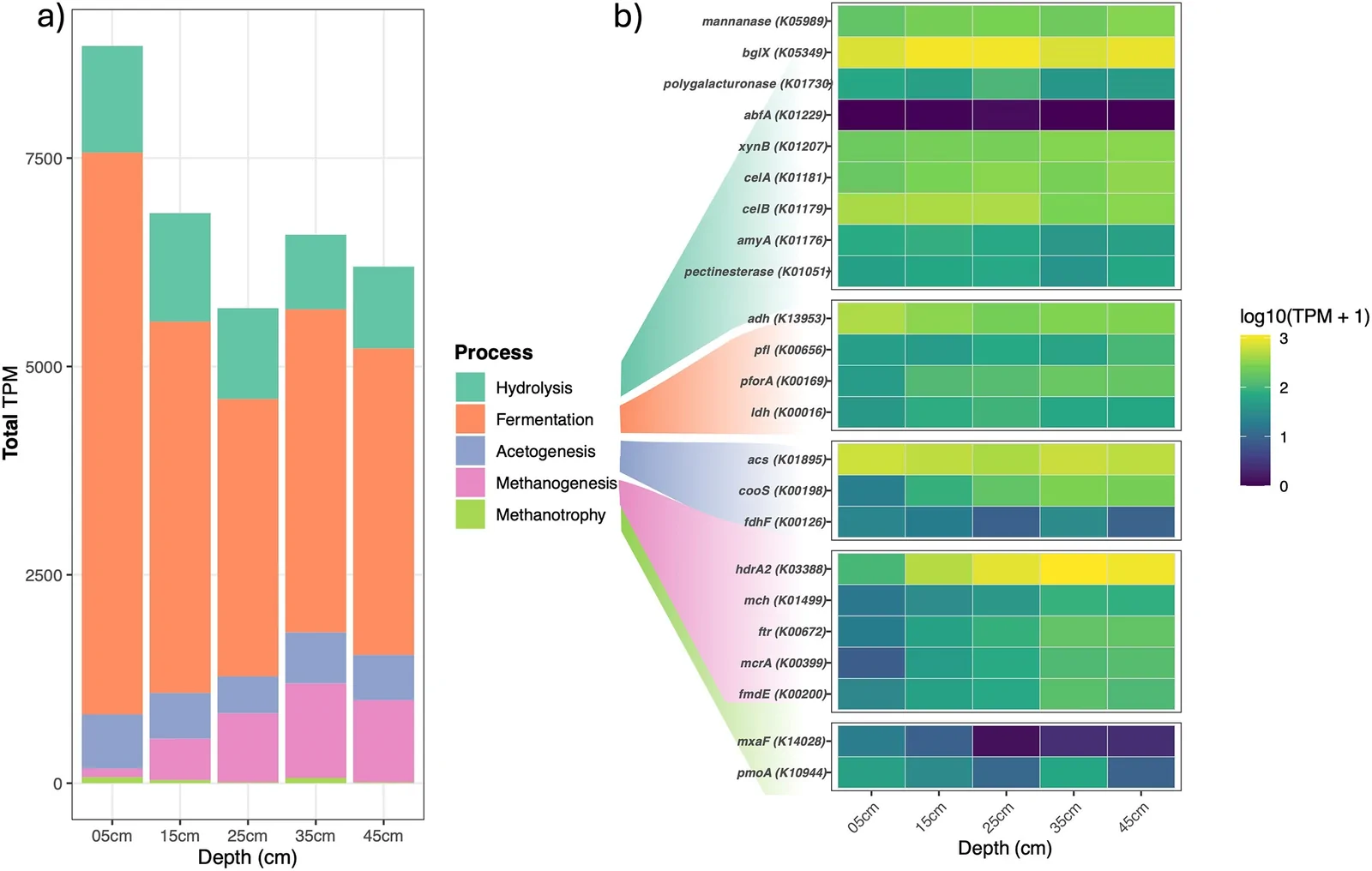
Depth-resolved functional profiles of anaerobic carbon cycling in peat soils. (a) Relative contributions of four major metabolic processes—hydrolysis, fermentation, acetogenesis, and methanogenesis—across five peat depths (5–45 cm), calculated from the summed TPM values of the selected KEGG Orthologs assigned to each functional category. (b) Heatmap showing the abundance of selected marker genes associated with hydrolysis, fermentation, acetogenesis, methanotrophy, and methanogenesis across the peat-depth gradient. Gene abundances are expressed as log₁₀(TPM + 1), accommodating zero values and reducing differences in scale among genes.

##### Hydrolysis

Hydrolysis-related marker genes were consistently detected across the peat profile, with most enzymes exhibiting moderate depth-dependent variation rather than sharp transitions in relative abundance (Fig. 6b). The dominant β-glucosidase *bglX* (K05349) increased from 750 TPM at 5 cm to around 1000 TPM at 15–25 cm, before declining to around 900 TPM in deeper layers. The xylanase *xynB* (K01207) showed a gradual increase with depth (from 201 to >300 TPM), whereas the endoglucanases *celA* and *celB* remained abundant but displayed modest fluctuations (e.g. *celA*: 183 to 277–315 to 335 TPM across 5–45 cm). In contrast, the α-amylase *amyA* (K01176) declined from around 70 TPM near the surface to 40–50 TPM at depth (Supplemental file S3). Additional CAZy hydrolases, including *abfA* (K01229), mannanase (K05989), pectinesterase (K01051), and polygalacturonase (K01730), were likewise detected throughout the profile, typically showing minor peaks at 15–25 cm followed by slight decreases at 35–45 cm.

The taxonomic affiliation of ORFs encoding hydrolysis-related KOs revealed a set of microbial contributors that were consistently detected across the peat profile. The cumulative metagenomic abundance assigned to the dominant taxa increased from ∼18% at 5 cm to 35%–40% at 25–35 cm, followed by a slight decline at 45 cm. The most abundant taxa included unclassified Acidobacteriota, Terriglobia, Verrucomicrobiota, and Acidobacteriaceae (Fig. S4).

##### Fermentation

Fermentation-related marker genes exhibited more pronounced depth-dependent variation as compared to hydrolytic functions (Fig. 6b). Alcohol dehydrogenase *adh* (K13953) showed a clear decline with depth, decreasing from 467 TPM at 5 cm to 240–294 TPM at mid-depth and stabilizing at 288 TPM at 45 cm. In contrast, lactate dehydrogenase *ldh* (K00016) remained relatively stable, fluctuating between 40 and 92 TPM across the profile. Other core fermentative enzymes displayed increasing trends toward deeper layers: pyruvate formate-lyase *pfl* (K00656) increased from 48 TPM at 5 cm to >100 TPM at 45 cm, and pyruvate: ferredoxin oxidoreductase *pforA* (K00169) rose from 48 TPM near the surface to 180+ TPM at 35–45 cm.

The taxonomic affiliation of ORFs encoding fermentation-related KOs indicated contributions from a diverse set of low-abundance but recurrent microbial lineages, including unclassified Pseudomonadota, Actinomycetota, Chloroflexota, Acidobacteriota, Verrucomicrobiota, and Deltaproteobacteria. Most contributing taxa showed relatively minor changes across the depth profile, whereas Chloroflexota and Terriglobia exhibited modest increases in deeper layers (Fig. S4).

##### Acetogenesis-related KOs

Acetogenesis-related marker genes displayed variable depth-dependent patterns (Fig. 6b). The acetyl-CoA synthetase gene *acs* (K01895), representing the terminal step of the Wood–Ljungdahl pathway, remained relatively stable across the profile, ranging from 650 TPM at 5 cm to 540–610 TPM at 35–45 cm. In contrast, the carbon monoxide dehydrogenase/acetyl-CoA synthase subunit *cooS* (K00198) showed a pronounced increase with depth, rising from 20 TPM at 5 cm to >250 TPM at 45 cm. The formate dehydrogenase gene *fdhF* (K00126) was detected at low abundance throughout the cores (typically <30 TPM) and exhibited no consistent depth trend. These patterns indicate that although core acetogenic potential remains broadly stable, certain upstream C₁-processing steps intensify under more anoxic conditions prevailing in deeper peat layers.

The taxonomic affiliation of ORFs encoding acetogenesis-related KOs revealed diverse contributors, collectively accounting for up to ∼8% of the total metagenomic taxonomic abundance. The dominant taxa included unclassified members of Acidobacteriota, Chloroflexota, Terriglobia, Verrucomicrobiota, Actinomycetota, and Hyphomicrobiales, along with archaeal groups such as Methanobacteriaceae and Euryarchaeota (Fig. S4).

It should be noted that the acetogenesis-related KOs analyzed here are not exclusive to acetogenic microorganisms and may also occur in other microbial groups, including methanogenic archaea. Archaeal-affiliated ORFs were primarily associated with K01895 (acetyl-CoA synthetase), whereas bacterial taxa, particularly Terriglobia, contributed increasingly to *cooS* (K00198) in deeper peat layers (Fig. S4).

##### Methanotrophy

Methanotrophy-related marker genes exhibited low but variable abundances across the peat profile (Fig. 6b). Methanol dehydrogenase *mxaF* (K14028) declined with depth, decreasing from 19 TPM at 5 cm to around 1 TPM at 35–45 cm. In contrast, the methane monooxygenase gene *pmoA* (K10944) decreased from 51 TPM at 5 cm to 10 TPM at 25 cm, followed by a pronounced peak of 62 TPM at 35 cm before declining again to 8.5 TPM at 45 cm.

The taxonomic affiliation of ORFs encoding methanotrophy-related KOs revealed a narrow group of contributors, whose cumulative contribution remained below 0.4% of the total metagenomic taxonomic abundance across all samples. Dominant taxa included *Methylocystis, Methylosinus, Methylocapsa*, and unclassified members of Methylocystaceae, Beijerinckiaceae, Gammaproteobacteria, and Hyphomicrobiales, representing predominantly aerobic methanotrophic bacteria. Most methanotrophic taxa exhibited decreasing relative abundance with depth, with the exception of Methylocystaceae and *Methylocystis*, which displayed a distinct enrichment at 35 cm, consistent with the transient increase in *pmoA* abundance observed at this depth (Fig. S4).

##### Methanogenesis

Methanogenesis-related marker genes showed a clear and consistent increase with depth (Fig. 6b). The heterodisulfide reductase gene *hdrA2* (K03388) rose sharply from 105 TPM at 5 cm to 497–829 TPM at 15–25 cm and peaked at 1134 TPM at 35 cm before remaining high at 985 TPM in the deepest layer. Upstream CO₂-reduction genes followed similar depth-dependent trends: *fmdE* (K00200) increased from 25 TPM near the surface to 113–143 TPM at 35–45 cm, and *ftr* (K00672) increased from 18 TPM to >160 TPM over the same interval. The key methane-forming enzyme *mcrA* (K00399) also increased strongly, from only 7.5 TPM at 5 cm to 133 TPM at 45 cm. Combined, these patterns indicate a substantial increase in methanogenic potential with depth, consistent with increasingly anoxic conditions in deeper peat layers.

The taxonomic affiliation of ORFs encoding methanogenesis-related KOs was dominated by a broad group of archaeal lineages whose cumulative contribution to the total metagenomic taxonomic abundance increased markedly with depth, rising from <0.1% in surface peat to ∼1% at 35–45 cm (Fig. S4). The majority of methanogenic potential was attributed to unclassified Methanomicrobia, with additional contributions from unclassified Methanoregulaceae, *Methanosarcina, Methanoregula*, and several unclassified Euryarchaeota-affiliated taxa. These unclassified archaeal lineages collectively accounted for the majority of methanogenesis-related ORFs across the peat profile, consistent with the marked increase in *mcrA* and other methanogenesis-associated marker genes with depth (Fig. S4).

#### Taxon-guided functional inference for representative microbial lineages

Several microbial lineages that were abundant or core members of the 16S rRNA gene community profiles were associated with a broad repertoire of metabolic functions (Supplemental file S9–S11). Surface-associated taxa such as *Aquisphaera* and *Roseiarcus* were linked to multiple hydrolysis-related KOs, including K05349 (β-glucosidase), K01176 (α-amylase), and K01179 (endoglucanase), suggesting a role in aerobic degradation of plant polymers.

Although *Methylocystis* and *Methylocapsa* are canonical alphaproteobacterial methanotrophs, the methanotrophy-associated KOs detected in this study (e.g. K10944 for particulate methane monooxygenase and K14028 for methanol dehydrogenase) were not assigned to these taxa in the current metagenomic dataset, but instead mapped primarily to unclassified lineages. In contrast, ORFs assigned to *Methylocystis* predominantly carried hydrolase-, fermentation-, and acetogenesis-related Kos (Supplemental file S11), including K00382 and K13953, indicating metabolic functions beyond classical methane oxidation.

*Clostridia* and *Thermoplasmata*, enriched in deeper, anoxic layers, were consistently associated with fermentation (e.g. K00656, K00382), acetogenesis (e.g. K01895, K00627), and additional stress-response-related KOs, reflecting their capacity for anaerobic degradation of organic compounds and adaptation to redox-limited environments. *Clostridia* in particular showed broad hydrolytic potential as well, with high abundance of K01179 (endoglucanase) and K05349 (β-glucosidase), indicating involvement in the breakdown of recalcitrant plant polymers prior to fermentation.

Among methanogenic archaea, Rice Cluster II was consistently abundant in the 16S rRNA gene dataset across peat depths, but no MAG was recovered for this lineage. At the broader taxonomic level, ORFs assigned to *Methanomicrobia* included methanogenesis-related KOs such as K00399 (methyl-coenzyme M reductase), K00200 (formylmethanofuran dehydrogenase), and K03388 (heterodisulfide reductase). These annotations support methanogenic potential within the community, but variation in metabolic capabilities within higher-level taxonomic groups limits lineage-specific functional inference. Consequently, these genes could not be confidently attributed to Rice Cluster II, and its metabolic potential could not be directly established from our metagenomic data.

Combined, these results highlight that although microbial communities show strong taxonomic stratification with depth, particularly at the genus level, there is considerable functional redundancy at the deeper layers (e.g. from 15 to 45 cm), where metabolically versatile taxa contribute to multiple pathways such as fermentation, acetogenesis, and methanogenesis.

## Discussion

### Robust vertical stratification across boreal peatlands

Consistent vertical stratification of microbial communities has been widely reported in northern peatlands (Andersen et al. 2013, Lin et al. 2014, Hausmann et al. 2018, Wang et al. 2024), reflecting strong environmental gradients in redox conditions, substrate quality, and nutrient availability. Our study confirms these patterns in boreal peatlands but extends them across a broader geographic range than most previous work, covering seven boreal peatlands building on 105 samples over five depth intervals. This high level of spatial and within-site replication allowed us to assess not only vertical but also spatial consistency of microbial stratification, which has been underexplored in previous studies that typically focused on individual peatlands or single cores (Lin et al. 2014, Wang et al. 2024). Both peat depth and peatland location significantly influenced microbial community composition, and their significant interaction indicated that the magnitude and composition of depth-related community shifts varied among peatlands. Thus, the observed vertical stratification was recurrent across sites but superimposed on substantial site-specific variation. The studied sites included both bogs and fens, yet the persistence of similar vertical patterns across peatlands suggests that shared and generic ecological processes associated with peat development exert a strong influence on microbial community assembly irrespective of peatland type. This consistency was further supported by the genome-resolved HQ/MQ MAG profiles, although archaeal groups such as Halobacteriota showed lower relative abundance in the MAG-derived profiles than in the 16S rRNA gene dataset. Vegetation composition, plant-derived organic matter inputs, hydrological conditions, and nutrient availability are all likely to contribute to these recurring depth-related patterns (Andersen et al. 2013, Lin et al. 2014)

We observed that surface communities (5 cm) had high representation of aerobic and facultative taxa associated with the degradation of fresh plant material, such as *Acidothermus, Bryobacter, Aquisphaera*, and *Roseiarcus*. These genera have been previously linked to polymer hydrolysis and fermentation under acidic and oxygenated conditions (Kulichevskaya et al. 2010, Bondoso et al. 2011, Kulichevskaya et al. 2014, St James et al. 2021). This near-surface stratification likely reflects oxygen diffusion dynamics and the rapid consumption of O_2_ and labile carbon near the surface, consistent with observations in Sphagnum-dominated peatlands (Tveit et al. 2013). In contrast, deeper peat layers (25–45 cm) were dominated by obligate anaerobes, including *Clostridia, Desulfobacca*, and archaeal lineages such as *Methanothrix, Methanoregula*, and *Rice Cluster II*, in agreement with findings from fen and bog environments with similar vertical redox gradients (Pester et al. 2012, Lin et al. 2014, Hausmann et al. 2018).

Alpha diversity declined sharply from 5 to 25 cm and remained low at greater depths, consistent with niches narrowing with dominance of metabolically specialized taxa. The decrease in microbial richness and diversity observed below the average water table does not unequivocally point to scarcity of resource or available energy as the sole factor creating less diverse microbial communities (Schlesinger and Bernhardt 2020). Instead, this pattern may reflect a specialized habitat where competition is limited, allowing a few resilient organisms, such as a dominant methanogen, to thrive. In fact, the presence of such specialized lineages suggests that low diversity could stem from competitive exclusion in environments where certain taxa are particularly well-adapted. This adaptation could be due to either recalcitrant organic matter availability or access to products of terminal decomposition pathways, and not merely the overall scarcity of resources.

In addition to taxonomic shifts, vertical changes in peat properties across the selection of peatlands investigated here have been reported. These include increasing bulk density and nitrogen content, and a gradual decrease in the C/N ratio with depth (Smeds et al. 2025). These trends are characteristic of progressive humification and compaction with depth (Broder et al. 2012, Leifeld et al. 2020), and have been linked to changes in microbial metabolic potential, particularly reduced energy yields from organic matter decomposition in deep peat layers (Blodau and Moore 2003, Beer and Blodau 2007). Consistent with this, our whole-metagenome functional profiling indicated a gradual shift from hydrolysis- and fermentation-dominated functions in surface peat toward proportionally greater methanogenic potential in deeper, anoxic layers. The alignment between microbial stratification and these chemical gradients supports the view that vertical niche partitioning in peatlands is not simply a reflection of redox zonation but also of evolving resource availability and stoichiometric constraints. In addition to physicochemical gradients, differences in vegetation composition and associated litter inputs may further contribute to shaping peat microbial communities, particularly in surface horizons where fresh plant-derived substrates are most abundant (Opelt et al. 2007, Bragina et al. 2014). The recurring depth-related gradients in peat physical and chemical properties likely impose broadly similar ecological filters across peatlands, while local environmental conditions modulate the magnitude and taxonomic expression of these depth-related responses.

### Core and keystone taxa define ecological structure along the peat profile

Across all sites, we observed a consistent core–transient community structure along the peat profile. Using a conservative prevalence threshold (≥80% occurrence), only a small proportion of genera were classified as core at each depth, whereas the majority of taxa occurred sporadically across samples. Notably, the proportion of core taxa remained relatively constant along the depth gradient, indicating that vertical stratification in peatlands does not necessarily involve an increasing prevalence of widespread taxa in deeper layers.

Despite this stable core–transient structure, community dominance increased with depth. The cumulative relative abundance of the five most dominant genera rose substantially from surface to deeper horizons, while α-diversity indices declined. This pattern suggests that depth-related environmental constraints favor a smaller number of highly adapted taxa that contribute disproportionately to community composition, without substantially expanding the set of taxa consistently present across samples.

Surface horizons exhibited higher richness and a larger pool of transient taxa, particularly among low-abundance genera (<1%), likely reflecting greater environmental heterogeneity and stronger temporal variability in redox conditions, and more diverse inputs of fresh plant-derived substrates (Shade and Handelsman 2012, Liu et al. 2022). In contrast, deeper layers were characterized by lower diversity but stronger dominance by a limited number of taxa adapted to energy-limited conditions and specialized metabolic niches. Together, these results indicate that decreasing diversity with depth reflects increasing ecological specialization and dominance rather than a shift toward a broader or more widespread core community.

Specifically, Rice Cluster II (family Methanoflorentaceae) emerged as a dominant and persistent archaeal lineage across intermediate and deep peat layers. Its presence as a core genus emphasizes the importance of amplicon sequencing in resolving ecologically important lineages that may not be recovered through genome-resolved metagenomic reconstruction. This lineage is phylogenetically closely related to *Methanomicrobia* and its significance in our study is supported by previous genomic and isotopic evidence to function as a hydrogenotrophic methanogen (Mondav et al. 2014), adapted to low-pH, oligotrophic peat environments (Genero 2017).

Co-occurrence network analysis further revealed a highly connected microbial module centered around Rice Cluster II, with strong positive associations to *Desulfobacca* and Subgroup 13 (Acidobacteriae). While the presence of *Desulfobacca*, a known sulfate reducer (Oude Elferink et al. 1999, Muyzer and Stams 2008), might at first suggest competition with methanogens, the low sulfate concentrations observed in our dataset (mean 0.003 mM) were below levels known to inhibit methanogenesis (Watson and Nedwell 1998). Under such conditions, sulfate-reducing bacteria may instead establish mutualistic fermentative or syntrophic interactions with the methanogens rather than competing for substrates. (Dar et al. 2008, Hu et al. 2019).

The functional and ecological distinction between core and keystone taxa has been discussed in microbial ecology (Liu et al. 2022). At the genus level, *Acidothermus, Aquisphaera*, and *Desulfobacca* emerged as keystone candidates based on high degree and closeness centrality, and low betweenness values in the co-occurrence network. Each of these taxa exhibited distinct ecological niches: *Acidothermus* and *Aquisphaera* are aerobic cellulolytic bacteria found in surface layers (Bondoso et al. 2011, St James et al. 2021), while *Desulfobacca* was more prevalent at depth, associated with anoxic metabolism.

### Shared metabolic potential across depth-stratified microbial communities

Vertical stratification in metabolic potential was consistently supported by both genome-resolved metabolic reconstruction from HQ/MQ MAGs and gene-centric whole-metagenome functional profiling. Aerobic or facultatively anaerobic taxa like *Acidothermus, Bryobacter*, and *Aquisphaera* were consistently detected in surface layers and are known to encode enzymes for carbohydrate degradation and partial fermentation (Reji and Zhang 2022). In deeper anoxic zones, recovered MAGs affiliated with Clostridia and Thermoplasmata encoded key pathways for fermentation and acetogenesis, whereas methanogenesis was primarily encoded by methanogenic archaea such as *Methanothrix* and *Methanoregula*. This layered metabolic organization supports a depth-resolved carbon flow, where complex polymers are processed into low-molecular-weight substrates that fuel terminal respiratory processes. This metabolic layering aligns with findings from Wang et al. (2024), who demonstrated that microbial communities and their functions are structured by vertical geochemical zones in a northern peatland, highlighting functional specialization despite low taxonomic diversity.

Notably, 16S rRNA gene data indicated both a substantial presence of methanogenic archaea below 15 cm depths and a dominance of hydrogenotrophic methanogenesis, largely driven by the high relative abundance of Rice Cluster II. In contrast, relatively few HQ/MQ archaeal MAGs were recovered compared with bacterial MAGs, whereas gene-centric ORF annotations captured a broader diversity of methanogenesis-related genes. This discrepancy likely reflects a combination of primer bias in 16S rRNA gene amplification (Parada et al. 2016), and technical challenges in assembling archaeal genomes from metagenomes, especially for low-abundance, high-GC, or genomically streamlined taxa (Tully et al. 2018, Bandla et al. 2023). These limitations highlight the complementary value of gene-centric whole-metagenome analyses, which in our case provided evidence of methanogenic potential beyond the recovered MAGs, although these functions could not be confidently assigned to Rice Cluster II. Similar gene-centric strategies have proven valuable in other peatland studies, where metagenomics has revealed previously undetected methanogenic lineages and functional diversity (Woodcroft et al. 2018).

Rice Cluster II formed the core of a particularly cohesive and depth-conserved module in our co-occurrence network, where it consistently co-occurred with *Desulfobacca* and Subgroup 13 (Acidobacteriae). Although no MAGs were recovered for Rice Cluster II, gene-centric annotation of ORFs assigned to *Methanomicrobia* (including Methanoflorentaceae-level affiliations where available) recovered hydrogenotrophic methanogenesis genes (*mcrA, hdr, fmd*), consistent with the genome of Candidatus Methanoflorentaceae, a known Rice Cluster II methanogen recovered from permafrost peatlands (Mondav et al. 2014). Our results are consistent with a potential role for Rice Cluster II-related methanogens in deep, anoxic peat layers across boreal peatlands in Sweden. This is also consistent with prior studies showing that Rice Cluster II methanogen are widespread in high methane-emitting ecosystems such as thawing permafrost wetlands and cold anoxic sediments globally (Godin et al. 2012, Hawkins et al. 2014, Mondav et al. 2014, Blake et al. 2015).

*Desulfobacca* was consistently detected in the 16S rRNA gene sequencing and co-occurred with methanogens and fermenters in deep peat, but no HQ/MQ MAGs were recovered for this genus. Its physiological profile, based on the type species *Desulfobacca acetoxidans*, confirms its capacity for dissimilatory sulfate reduction (Stams and Oude Elferink 2015, Oude Elferink et al. 1999). Although sulfate-reduction marker genes (*dsrA*/*dsrB*) were detected in the metagenomic ORF dataset, they were not taxonomically assigned to *Desulfobacca*, preventing direct functional attribution to this lineage. Given the low *in situ* sulfate concentrations, *Desulfobacca* may instead rely on alternative electron acceptors or function syntrophically in acetate oxidation, consistent with its ecological behavior in other wetland ecosystems (Hamovit et al. 2025).

Acidobacteriota were well represented in our MAGs, including members of the class Terriglobia and other undefined groups, although none could be confidently assigned to Subgroup 13. Several MAGs encoded CAZymes and genes associated with fermentation, supporting their involvement in oligosaccharide degradation and organic matter mineralization. Such metabolic traits are consistent with adaptation to the complex and progressively humified organic matter characteristic of deeper peat horizons. These shared metabolic features, including acetate, formate, and hydrogen turnover, suggest a stable syntrophic network in which tightly coordinated substrate handoffs enable thermodynamically constrained carbon transformations. Similar functionally integrated modules were described by Richy et al. (2024), who reported that Acidobacteriota dominated carbohydrate degradation pathways and co-occurred with hydrogenotrophic methanogens in depth-stratified MAG modules in acidic bogs. Wang et al. (2024) likewise highlighted Acidobacteria as important contributors to deep-peat carbon processing, forming redox-structured associations with methanogenic archaea across boreal peatlands.

Beyond classical prokaryotic interactions, other underexplored layers of microbial complexity may influence peatland carbon cycling. While not identified as core or keystone taxa, several MAGs affiliated with Myxococcota were present and occasionally abundant, particularly in deep layers. These predatory or saprotrophic bacteria may contribute to microbial biomass turnover or secondary carbon degradation, especially under low-energy conditions (Reji and Zhang 2022, Wang et al. 2024). In addition, viral dynamics, shown to modulate Acidobacteria and carbon turnover in bogs (Richy et al. 2024), were not characterized in our study, but likely play an important role in shaping microbial interactions, stability, and carbon fate. Together, these components underscore a level of trophic complexity and regulation that extends beyond what can be captured by genome-centric inference alone.

Moreover, while our results highlight depth-resolved patterns in both microbial composition and functional gene profiles, they also point to a noteworthy degree of functional overlap across deeper peat layers. Between 15 and 45 cm, genes related to fermentation, acetogenesis, and both hydrogenotrophic and acetoclastic methanogenesis were consistently detected, albeit with varying relative abundances. This indicates that metabolically versatile taxa may contribute to overlapping pathways across these depths, suggesting that despite taxonomic turnover, microbial communities retain functional redundancy that may buffer key ecosystem processes under changing environmental conditions (Woodcroft et al. 2018, Song et al. 2023). Alternatively, the observed overlap might also be partly explained by passive microbial transport along the peat profile under fluctuating water-table conditions. Together, the complementary genome-resolved and gene-centric analyses indicate that taxonomic turnover with depth is accompanied by a gradual reorganization of metabolic potential rather than an abrupt replacement of ecosystem functions.

## Conclusions

Microbial communities exhibited recurring depth-dependent organization across the seven boreal bog and fen ecosystems, despite substantial geographic distances and variation in climate, nutrient regime, and hydrological conditions. Although the magnitude and composition of depth-related shifts varied among peatlands, these recurring patterns suggest that vertical environmental gradients associated with peat development and hydrological conditions exert a broad influence on microbial community assembly across boreal peatlands.

The combined taxonomic, genome-resolved, and gene-centric analyses revealed a gradual transition from aerobic surface communities to increasingly anaerobic microbial assemblages with depth, while simultaneously demonstrating that key carbon-cycling functions remain broadly conserved across deeper peat horizons. These findings support the existence of recurring methane-associated microbial networks that appear to represent a common organizational feature of boreal peat ecosystems.

Although direct microbial activity was not measured in the present study, the observed consistent taxonomic and functional organization suggests that depth-resolved microbial assembly appears to be a recurring ecological feature of boreal peat ecosystems. Incorporating this vertical organization into future studies and ecosystem models may improve predictions of methane cycling and the microbial responses of peatlands to hydrological change and climate-driven environmental perturbations.

## Supplementary Material

fiag111_Supplemental_Files

## Data Availability

The raw metagenomic sequencing data and metagenome-assembled genomes (MAGs) generated in this study have been deposited in the NCBI Sequence Read Archive (SRA) under BioProject PRJNA1048504 (BioSample accession SAMN38645172). All other data supporting the findings of this study are included within the article and its Supplementary material files.

## References

[bib1] Andersen R, Chapman SJ, Artz RRE. Microbial communities in natural and disturbed peatlands: a review. Soil Biol Biochem. 2013;57:979–94. 10.1016/j.soilbio.2012.10.003.

[bib2] Bandla A, Mukhopadhyay S, Mishra S et al. Genome-resolved carbon processing potential of tropical peat microbiomes from an oil palm plantation. Sci Data. 2023;10:373. 10.1038/s41597-023-02267-z. PMC10250450 37291271

[bib3] Beer J, Blodau C. Transport and thermodynamics constrain belowground carbon turnover in a northern peatland. Geochim Cosmochim Acta. 2007;71:2989–3002. 10.1016/j.gca.2007.03.010.

[bib4] Berry D, Widder S. Deciphering microbial interactions and detecting keystone species with co-occurrence networks. Front Microbiol. 2014;5:219. 10.3389/fmicb.2014.00219 PMC4033041 24904535

[bib5] Blake LI, Tveit A, Øvreås L et al. Response of methanogens in Arctic sediments to temperature and methanogenic substrate availability. PLoS One. 2015;10:e0129733. 10.1371/journal.pone.0129733. PMC4471053 26083466

[bib6] Blodau C, Moore TR. Micro-scale CO_2_ and CH_4_ dynamics in a peat soil during a water fluctuation and sulfate pulse. Soil Biol Biochem. 2003;35:535–47. 10.1016/S0038-0717(03)00008-7.

[bib7] Bondoso J, Albuquerque L, Nobre M et al. Aquisphaera giovannonii gen. nov., sp. nov., a planctomycete isolated from a freshwater aquarium. Int J Syst Evol Microbiol. 2011;61:2844–50. 10.1099/ijs.0.027474-0. 21239565

[bib8] Bragina A, Oberauner-Wappis L, Zachow C et al. The Sphagnum microbiome supports bog ecosystem functioning under extreme conditions. Mol Ecol. 2014;23:4498–510. 10.1111/mec.12885. 25113243

[bib9] Broder T, Blodau C, Biester H et al. Peat decomposition records in three pristine ombrotrophic bogs in southern Patagonia. Biogeosciences. 2012;9:1479–91. 10.5194/bg-9-1479-2012.

[bib10] Callahan BJ, McMurdie PJ, Rosen MJ et al. DADA2: high-resolution sample inference from Illumina amplicon data. Nat Methods. 2016;13:581–3. 10.1038/nmeth.3869. PMC4927377 27214047

[bib11] Chaumeil P-A, Mussig AJ, Hugenholtz P et al. GTDB-Tk v2: memory friendly classification with the genome taxonomy database. Bioinformatics. 2022;38:5315–6. 10.1093/bioinformatics/btac672. PMC9710552 36218463

[bib12] Chklovski A, Parks DH, Woodcroft BJ et al. CheckM2: a rapid, scalable and accurate tool for assessing microbial genome quality using machine learning. Nat Methods. 2023;20:1203–12. 10.1038/s41592-023-01940-w. 37500759

[bib13] Clymo RS . A High-Resolution Sampler of Surface Peat. Funct Ecol. 1988;2:425–31. 10.2307/2389416.

[bib14] Dar SA, Kleerebezem R, Stams AJ et al. Competition and coexistence of sulfate-reducing bacteria, acetogens and methanogens in a lab-scale anaerobic bioreactor as affected by changing substrate to sulfate ratio. Appl Microbiol Biotechnol. 2008;78:1045–55. 10.1007/s00253-008-1391-8 PMC2271084 18305937

[bib15] Frolking S, Talbot J, Jones MC et al. Peatlands in the Earth’s 21st century climate system. Environ Rev. 2011;19:371–96. 10.1139/a11-014.

[bib16] Genero MM . Microbial Communities in Boreal Peatlands: responses to Climate Change and Atmospheric Nitrogen and Sulfur Depositions. Linköping, Sweden: Linköping University Electronic Press, 2017 .

[bib17] Godin A, McLaughlin JW, Webster KL et al. Methane and methanogen community dynamics across a boreal peatland nutrient gradient. Soil Biol Biochem. 2012;48:96–105. 10.1016/j.soilbio.2012.01.018.

[bib18] Hamovit N, RoyChowdhury T, Akob DM et al. Comparative assessment of a restored and natural wetland using ^13^C-DNA SIP reveals a higher potential for methane production in the restored wetland. Appl Environ Microb. 2025;91:e02161–02124. 10.1128/aem.02161-24. PMC11921397 39912641

[bib19] Hausmann B, Pelikan C, Herbold CW et al. Peatland Acidobacteria with a dissimilatory sulfur metabolism. ISME J. 2018;12:1729–42. 10.1038/s41396-018-0077-1. PMC6018796 29476143

[bib20] Hawkins AN, Johnson KW, Bräuer SL. Southern Appalachian peatlands support high archaeal diversity. Microb Ecol. 2014;67:587–602. 10.1007/s00248-013-0352-7. 24419541

[bib21] Haynes W . Benjamini–Hochberg method. In: Dubitzky W, Wolkenhauer O, Cho K-H, Yokota H (eds.), Encyclopedia of Systems Biology. New York, NY: Springer, 2013, 78. 10.1007/978-1-4419-9863-7_1215

[bib22] Herlemann DPR, Labrenz M, Jürgens K et al. Transitions in bacterial communities along the 2000 km salinity gradient of the Baltic Sea. ISME J. 2011;5:1571–9. 10.1038/ismej.2011.41. PMC3176514 21472016

[bib23] Hu H, Wang B, Bravo A et al. Shifts in mercury methylation across a peatland chronosequence: from sulfate reduction to methanogenesis and syntrophy. J Hazard Mater. 2020;387:121967. 10.1016/j.jhazmat.2019.121967. 31901845

[bib24] Hug LA, Baker BJ, Anantharaman K et al. A new view of the tree of life. Nat Microbiol. 2016;1:16048. 10.1038/nmicrobiol.2016.48. 27572647

[bib25] Kulichevskaya IS, Danilova OV, Tereshina VM et al. Descriptions of Roseiarcus fermentans gen. nov., sp. nov., a bacteriochlorophyll a-containing fermentative bacterium related phylogenetically to alphaproteobacterial methanotrophs, and of the family Roseiarcaceae fam. nov. Int J Syst Evol Microbiol. 2014;64:2558–65. 10.1099/ijs.0.064576-0. 24812364

[bib26] Kulichevskaya IS, Suzina NE, Liesack W et al. Bryobacter aggregatus gen. nov., sp. nov., a peat-inhabiting, aerobic chemo-organotroph from subdivision 3 of the Acidobacteria. Int J Syst Evol Microbiol. 2010;60:301–6. 10.1099/ijs.0.013250-0. 19651730

[bib27] Leifeld J, Klein K, Wüst-Galley C. Soil organic matter stoichiometry as indicator for peatland degradation. Sci Rep. 2020;10:7634. 10.1038/s41598-020-64275-y. PMC7203203 32376905

[bib28] Lin X, Tfaily MM, Green SJ et al. Microbial metabolic potential for carbon degradation and nutrient (nitrogen and phosphorus) acquisition in an ombrotrophic peatland. Appl Environ Microb. 2014;80:3531–40. 10.1128/AEM.00206-14. PMC4018864 24682299

[bib29] Liu T, Goux X, Calusinska M et al. Analyzing microbial core communities, rare species, and interspecies interactions can help identify core microbial functions in anaerobic degradation. Assess Microbiol Health Ecosyst. 2022;127–57. 10.1002/9781119678304 10.1002/9781119678304.ch6.

[bib30] Lolu AJ, Ahluwalia AS, Sidhu MC et al. Carbon sequestration and storage by wetlands: implications in the climate change scenario. Restor Wetl Ecosyst Traj Sustain Environ. 2020;45–58.

[bib31] Mondav R, Woodcroft BJ, Kim E-H et al. Discovery of a novel methanogen prevalent in thawing permafrost. Nat Commun. 2014;5:3212. 10.1038/ncomms4212. 24526077

[bib32] Muyzer G, Stams AJM. The ecology and biotechnology of sulphate-reducing bacteria. Nat Rev Micro. 2008;6:441–54. 10.1038/nrmicro1892. 18461075

[bib33] Opelt K, Chobot V, Hadacek F et al. Investigations of the structure and function of bacterial communities associated with Sphagnum mosses. Environ Microbiol. 2007;9:2795–809. 10.1111/j.1462-2920.2007.01391.x. 17922763

[bib34] Oude Elferink SJ, Akkermans-van Vliet WM, Bogte JJ et al. Desulfobacca acetoxidans gen. nov., sp. nov., a novel acetate-degrading sulfate reducer isolated from sulfidogenic granular sludge. Int J Syst Bacteriol. 1999;49:345–50. 10.1099/00207713-49-2-345 10319454

[bib35] Parada AE, Needham DM, Fuhrman JA. Every base matters: assessing small subunit rRNA primers for marine microbiomes with mock communities, time series and global field samples. Environ Microbiol. 2016;18:1403–14. 10.1111/1462-2920.13023. 26271760

[bib36] Pester M, Knorr K-H, Friedrich MW et al. Sulfate-reducing microorganisms in wetlands—fameless actors in carbon cycling and climate change. Front Microbio. 2012;3:2012. 10.3389/fmicb.2012.00072. PMC3289269 22403575

[bib37] Quast C, Pruesse E, Yilmaz P et al. The SILVA ribosomal RNA gene database project: improved data processing and web-based tools. Nucleic Acids Res. 2013;41:D590–6. 10.1093/nar/gks1219. PMC3531112 23193283

[bib38] Reji L, Zhang X. Genome-resolved metagenomics informs the functional ecology of uncultured acidobacteria in redox oscillated *Sphagnum*peat. mSystems. 2022;7:e00055–00022. 10.1128/msystems.00055-22. PMC9599518 36036503

[bib39] Richy E, Cabello-Yeves PJ, Hernandes-Coutinho F et al. How microbial communities shape peatland carbon dynamics: new insights and implications. Soil Biol Biochem. 2024;191:109345. 10.1016/j.soilbio.2024.109345.

[bib40] Salimi S, Almuktar SAAAN, Scholz M. Impact of climate change on wetland ecosystems: a critical review of experimental wetlands. J Environ Manage. 2021;286:112160. 10.1016/j.jenvman.2021.112160. 33611067

[bib41] Schlesinger WH, Bernhardt ES. Wetland ecosystems. Biogeochemistry. 2020;249–91. 10.1016/B978-0-12-814608-8.00007-4.

[bib42] Shade A, Handelsman J. Beyond the Venn diagram: the hunt for a core microbiome. Environ Microbiol. 2012;14:4–12. 10.1111/j.1462-2920.2011.02585.x. 22004523

[bib43] Shen L, Peng S, Zhang Z et al. The large role of declining atmospheric sulfate deposition and rising CO_2_concentrations in stimulating future wetland CH_4_emissions. Sci Adv. 2025;11:eadn1056. 10.1126/sciadv.adn1056. PMC11797542 39908374

[bib44] Sinclair L, Osman OA, Bertilsson S et al. Microbial community composition and diversity via 16S rRNA gene amplicons: evaluating the illumina platform. PLoS One. 2015;10:e0116955. 10.1371/journal.pone.0116955. PMC4315398 25647581

[bib45] Sipos R, Székely AJ, Palatinszky M et al. Effect of primer mismatch, annealing temperature and PCR cycle number on 16S rRNA gene-targetting bacterial community analysis. FEMS Microbiol Ecol. 2007;60:341–50. 10.1111/j.1574-6941.2007.00283.x. 17343679

[bib46] Smeds J, Ehnvall B, Liu T et al. Effect of restoration on physical and chemical peat properties in previously drained boreal peatlands. Ecosystems. 2025;28:45. 10.1007/s10021-025-00991-8.

[bib47] Song T, Liu Y, Kolton M et al. Porewater constituents inhibit microbially mediated greenhouse gas production (GHG) and regulate the response of soil organic matter decomposition to warming in anoxic peat from a Sphagnum-dominated bog. FEMS Microbiol Ecol. 2023;99:fiad060. 10.1093/femsec/fiad060 37280172

[bib48] Stams AJM, Oude Elferink SJWH. Desulfobacca. In: Whitman WB (ed.), Bergey’s Manual of Systematics of Archaea and Bacteria. Hoboken, NJ: John Wiley & Sons, Inc, 2015, 1–2.

[bib49] St James AR, Yavitt JB, Zinder SH et al. Linking microbial Sphagnum degradation and acetate mineralization in acidic peat bogs: from global insights to a genome-centric case study. Isme j. 2021;15:293–303. 10.1038/s41396-020-00782-0 PMC7852519 32951020

[bib50] Tamames J, Puente-Sánchez F. SqueezeMeta, a highly portable, fully automatic metagenomic analysis pipeline. Front Microbiol. 2019;9:3349 10.3389/fmicb.2018.03349 PMC6353838 30733714

[bib51] Tully BJ, Graham ED, Heidelberg JF. The reconstruction of 2631 draft metagenome-assembled genomes from the global oceans. Sci Data. 2018;5:170203. 10.1038/sdata.2017.203. PMC5769542 29337314

[bib52] Tveit A, Schwacke R, Svenning MM et al. Organic carbon transformations in high-Arctic peat soils: key functions and microorganisms. ISME J. 2013;7:299–311. 10.1038/ismej.2012.99. PMC3554415 22955232

[bib53] Wang H-Y, Yu Z-G, Zhou F-W et al. Microbial communities and functions are structured by vertical geochemical zones in a northern peatland. Sci Total Environ. 2024;950:175273. 10.1016/j.scitotenv.2024.175273. 39111416

[bib54] Watson A, Nedwell DB. Methane production and emission from peat: the influence of anions (sulphate, nitrate) from acid rain. Atmos Environ. 1998;32:3239–45. 10.1016/S1352-2310(97)00501-3.

[bib55] Weinstein MM, Prem A, Jin M et al. FIGARO: an efficient and objective tool for optimizing microbiome rRNA gene trimming parameters. Biorxiv. 2019;610394.

[bib56] Woodcroft BJ, Singleton CM, Boyd JA et al. Genome-centric view of carbon processing in thawing permafrost. Nature. 2018;560:49–54. 10.1038/s41586-018-0338-1. 30013118

[bib57] Zhou X, Zhang Z, Tian L et al. Microbial communities in peatlands along a chronosequence on the Sanjiang Plain, China. Sci Rep. 2017;7:9567. 10.1038/s41598-017-10436-5. PMC5575048 28852134

