# Supplementary material for: Depth-stratified boreal peatland microbiomes reveal recurring community organization and methane-cycling potential
Source: FEMS Microbiol Ecol. 2026 Oct 1;102(10):fiag111. doi: 10.1093/femsec/fiag111 (PMC13643928; doi:10.1093/femsec/fiag111)
Supplement: Supplementary file 1 [file fiag111_supplemental_files.zip › 20261001_Supplemental Figures and Tables.docx]

Figure S1. Non-metric multidimensional scaling (NMDS) of microbial community composition across peatlands. Colored by peatland location.

Figure S2. Pearson correlation analysis between environmental variables. Cells in the matrix are color-coded according to the Pearson correlation coefficient values, ranging from -1 to 1. The color scale to the right of the heatmap indicates the strength and direction of the correlation: a value of 1 (dark blue) represents a perfect positive correlation; a value of 0 (white) indicates no correlation; and a value of -1 (dark red) represents a perfect negative correlation. Variables included in the analysis: CH₄ (porewater methane concentration, ppm), BD (bulk density, g cm⁻³), Organic matter content (%), Acetate concentration (mg/L), Sulfate concentration (mg/L), C% (carbon content, %), N% (nitrogen content, %), C/N (carbon-to-nitrogen ratio), pH (porewater pH), GWL_Ave (annual average groundwater table level, cm), and GWL_std (standard deviation of groundwater level during the monitoring period, cm).

Figure S3. Alpha diversity metrics (Observed richness, Shannon, and Simpson) across peat depths (5–45 cm) based on 16S rRNA gene amplicon data. Violin and boxplots show the distribution of diversity values for replicate samples within each depth interval. Pairwise differences between depths were evaluated using Wilcoxon rank-sum tests, with significance indicated by p-value thresholds (p < 0.05: *, p < 0.01: **, p < 0.001: ***, p < 0.0001: ****). “ns” indicates non-significant differences. Overall differences among depths for each diversity index were assessed using Kruskal–Wallis tests, with the global p-value shown at the top of each panel.

Figure S4. Taxonomic affiliation of metagenomic ORFs assigned to KEGG Orthologs (KOs) associated with five key metabolic processes: hydrolysis, fermentation, acetogenesis, methanotrophy, and methanogenesis. Bars represent the relative metagenomic abundance of taxa represented by ORFs assigned to each functional category. Relative abundances are shown without rescaling within individual functional categories. Only taxa represented by at least one annotated ORF are shown.

| *Peatland* | *Peatland code* | *Region* | *Peatland type* | *Latitude and longitude* |
| --- | --- | --- | --- | --- |
| Ånnsjön mire | AN_1_3; AN_4_6 | Jämtland | Fen | 63°18'49"N, 12°31'35"E |
| Öjsjömyrarna | OM | Jämtland | Fen | 63°27'34"N, 15°05'35"E |
| Mossaträsk  Stensjöflon | MT  SF | Västernorrland  Västernorrland | Fen  Fen | 63°49'20"N, 17°18'10"E  63°15'04"N, 16°28'43"E |
| Bredsjömossen | BM | Östergötland | Fen | 58°46'53"N, 15°12'53"E |
| Anderstorps Stormosse | AS | Jönköping | Bog | 57°19'34"N, 13°36'42"E |
| Store Mosse | SM | Jönköping | Bog | 57°16'57"N, 13°55'18"E |

Table S1. Location of the peatland sampling sites.
